# Genomic landscape of Mexican patients with maturity onset diabetes of the young: beyond mutations in MODY-known genes

**DOI:** 10.3389/fendo.2026.1887861

**Published:** 2026-07-01

**Authors:** Alberto Moscona-Nissan, Daniel Marrero-Rodríguez, Sergio Andonegui-Elguera, Eduardo Salif Luna-Ávila, Florencia Martínez-Mendoza, Sandra Vela-Patiño, Itzel Ramírez-Ramos, Silvia Hinojosa-Alvarez, Jesus Hernandez-Perez, Rocio A. Chavez-Santoscoy, Sophia Mercado-Medrez, Kapy S. León-Wu, Regina De Miguel-Ibáñez, Moisés Mercado, Keiko Taniguchi-Ponciano, Aldo Ferreira-Hermosillo

**Affiliations:** 1 Unidad de Investigación Médica en Enfermedades Endocrinas, Hospital de Especialidades, Centro Médico Nacional Siglo XXI, Instituto Mexicano del Seguro Social, Mexico City, Mexico; 2 Escuela de Ingeniería y Ciencias, Tecnológico de Monterrey, Monterrey, Mexico

**Keywords:** diabetes, genetics, Latin America, metabolism, MODY

## Abstract

**Introduction:**

Maturity Onset Diabetes of the Young (MODY) remains an underdiagnosed condition with remarkable genetic variability across populations. While diagnostic tools are based on Caucasian cohorts, Whole Exome Sequencing (WES) studies are needed to identify new genes in non-Caucasians, as up to 77% of patients do not harbor variants of significance in MODY-known genes. No WES studies have addressed the genomic landscape of MODY beyond its canonical genes in Latino populations. We aimed to characterize the genomic landscape of MODY through WES in a Mexican cohort, comparing cases with type 2 diabetes mellitus (T2DM) patients and healthy controls (HC).

**Methods:**

WES was performed in 17 patients with MODY, 17 with T2DM and 17 HC. We compared the single nucleotide variant landscape across groups in MODY-known genes and searched for genetic variants with differential enrichment across groups.

**Results:**

MODY genes used for routine diagnosis showed low discrimination utility, as patients with MODY, T2DM and HC harbored genetic variants in MODY-known genes at similar frequencies in most cases. We found 14 genes with variants capable of distinguishing MODY from T2DM and HC. Variants in genes such as *MAP2K3*, *SYT15*, *KCNJ12*, *PEX5*, and *TPTE* were found in 75-100% of MODY cases while absent in T2DM and HC. Enrichment analysis revealed involvement in synaptic vesicle trafficking, insulin/IGF pathway-mitogen activated protein kinase kinase/MAPK, and insulin/IGF pathway-protein kinase B/AKT signaling.

**Discussion:**

MODY presents a complex genetic architecture in the Mexican population. Besides improving our understanding of glycemic regulation pathways, identified genes may serve as diagnostic biomarkers.

## 1 Introduction

Maturity-onset diabetes of the young (MODY) comprises a clinically heterogeneous group of monogenic forms of diabetes characterized by autosomal dominant inheritance, early onset, and absence of pancreatic beta-cell autoimmunity (1). MODY is associated with distinct mutations in genes involved in pancreatic beta-cell differentiation, insulin secretion, and glucose metabolism (2). The most frequently affected genes include those encoding hepatocyte nuclear factor 1α (*HNF1A*), hepatocyte nuclear factor 4α (*HNF4A*), and glucokinase (*GCK*). Mutations in other genes have been described but are infrequent (2, 3).

Approximately 3% of patients with MODY are diagnosed correctly, while the rest are mistakenly classified as type 1 diabetes mellitus and type 2 diabetes mellitus (T2DM) in 36% and 51% of cases, respectively (4, 5). MODY misdiagnosis remains widespread, as physicians face challenges such as overlapping clinical phenotypes between MODY and other diabetes forms (6). Certain MODY subtypes such as *HNF1A*- and *HNF4A*-MODY are associated with an increased risk of micro- and macrovascular complications, while *GCK*-MODY is associated with a mild clinical course and lower complication rates. Furthermore, other rare MODY subtypes are associated with additional comorbidities such as kidney and urogenital tract malformations, exocrine pancreas dysfunction, and neurological manifestations (7).

Establishing an accurate diagnosis is critical to offer tailored therapies, genetic counseling, and prevent complications (6). There is remarkable genetic variability in patients with MODY across world populations. Currently, MODY diagnosis relies on its clinical features and genetic tools developed in Caucasian cohorts, while there is limited information on the genetics in other populations. This gap is highlighted by the fact that up to 77% of Middle Eastern patients with MODY lack variants of significance in known genes, while other populations have genetic confirmation rates as low as 6.6% (8–10).

In Latino populations, no whole exome sequencing (WES) studies have addressed the genetic landscape of MODY beyond its known genes. In Mexico, despite the high prevalence of diabetes (18.3%) associated with a 75% prevalence of overweight and obesity, few studies have addressed MODY genetics (11). Latino populations are highly affected by diabetes, yet remain underrepresented in genetic studies, and present a complex genetic admixture that could shape the genomic landscape of MODY (12). These factors highlight the need to further study the genomic aspects of MODY and identify new genes involved in its pathogenesis. The aim of this study was to characterize the genomic landscape in Mexican patients with MODY through WES, comparing them to individuals with Type 2 Diabetes Mellitus (T2DM), and healthy controls (HC).

## 2 Materials and methods

### 2.1 Clinical features, selection criteria and definitions

We enrolled 51 participants divided into three groups each having 17 subjects: MODY, T2DM and HC. All MODY and T2DM patients were treated and followed in the outpatient clinics of Centro Médico Nacional Siglo XXI, the largest tertiary care facility in Mexico. All subjects signed an informed consent to participate. The study was approved by the scientific and ethics committees (R-2023-3601-003, R-2023-3601-212) and conducted in accordance with research laws and the Helsinki declaration.

Patients with MODY were diagnosed based on an Exeter score ≥36%, low or null insulin requirements (<1 U/Kg/day), preserved beta-cell function (serum C-peptide >1ng/mL) and lack of T1DM autoantibodies. The T2DM group included adult patients diagnosed according to the American Diabetes Association criteria (HbA1c ≥6.5%, fasting plasma glucose ≥126 mg/dL, a 2-h plasma glucose ≥200 mg/dL during oral glucose tolerance test, or presence of classical symptoms of hyperglycemia or hyperglycemic crisis, with a random glucose ≥200 mg/dL) with <10 years of diagnosis, regardless of type of treatment and HbA1c levels, and an Exeter score ≤5%. HC comprised adult volunteers, without personal or parental history of diabetes or cardiometabolic diseases such as hypertension and dyslipidemia. All subjects were from the central region of Mexico, and none had any familial relationship with each other. We retrieved clinical, biochemical, and anthropometric information from medical records. We calculated the Exeter score using the MODY probability Calculator for all MODY and T2DM cases diagnosed under 35 years (available at: https://www.diabetesgenes.org/exeter-diabetes-app/) (**Table 1**). We increased the Exeter-score cut-off value to ≥36% above the original 25%, since this value proved a better diagnostic performance in non-Caucasian populations with mixed genetic admixture (13, 14). A sensitivity and specificity of 98% and 92% for this score have been reported using a 30% cut-off point in the Brazilian population, which could be the closest reference to our population (15).

**Table 1:** Sociodemographic and clinical data of included participants.

	MODY (n=17)	Type 2 diabetes (n=17)	Healthy controls (n=17)	P-value
Sex				
Male (%) Female (%)	4 (24) 13 (76)	4 (24) 13 (76)	9 (53) 8 (47)	0.11
Age at diagnosis				
mean (SD)	21.2 (5.7)	43.5 (10.1	NA	<0.001
Age at evaluation				
mean (SD)	34.1 (10.7)	52.2 (11.1	25.5 (3.4)	<0.001
Biochemical evaluation				
Mean HbA1c % (SD) Mean HbA1c (SD), mmol/mol Under treatment goals HbA1c <7 (%)	7.12 (1.1) 54.3(12.0) 8 (47.0)	7.0 (1.0) 52 (10.9) 9 (52.9)	NA NA NA	0.393 0.393 0.73
Parental history of diabetes				
n (%)	15 (88.2)	9 (52.9)	0 (0)	<0.001 (All) 0.023 (MODY vs T2DM)
Comorbidities				
Mean BMI (SD), kg/m^2^ Waist circumference, cm Overweight (%) Obesity (%) Normal weight (%) Underweight (%) Hypertension (%) Dyslipidemia (%)	25.1 (4.7) 88.0 (12.0) 4 (23.5) 2 (11.7) 10 (58.8) 1 (5.8) 1 (5.8) 13 (76.4)	37.3 (10.4) 109.5 (19.0) 3 (17.6) 12 (70.5) 1 (5.8) 1 (5.8) 13 (76.4) 5 (27.7)	24.2 (3.0) 80.8 (9.1) 6 (35.2) 1 (5.8) 9 (52.9) 1 (5.8) 0 (0) 0 (0)	<0.001 <0.001 0.48 <0.001 0.002 1.0 <0.001 <0.001
Calculated Exeter score				
Mean (SD) Min - Max	63.5 (14.3) 35.8 - 75.5	4.6 (0) 4.6 - 4.6	NA NA	<0.001
Current treatment				
Insulin (%) Metformin (%) Sulfonylureas (%) DPP-4 inhibitors (%) GLP-1 agonists (%) SGLT2 inhibitors (%) Thiazolidinediones (%) Diet and exercise (%)	7 (41.1) 10 (58.8) 6 (35.2) 3 (17.6) 3 (17.6) 3 (17.6) 1 (5.9) 1 (5.9)	7 (41.1) 14 (82.3) 1 (5.8) 6 (35.2) 8 (47.0) 8 (47.0) 2 (11.7) 0 (0)	NA NA NA NA NA NA NA NA	1.00 0.13 0.033 0.24 0.066 0.066 0.36 0.31
MODY type suspected				
GCK-MODY HNF1A-MODY HNF1B-MODY PAX4-MODY HNF1A/B-MODY dual suspicion GCK/HNF1A-MODY dual suspicion	1 (5.9) 8 (47.0) 1 (5.9) 2 (11.7) 4 (23.5) 1 (5.9)	NA	NA	NA

BMI, Body Mass Index; DPP-4, Dipeptidyl Peptidase 4; Estimated average glucose, equivalent of glycated hemoglobin in mg/dL; GLP-1, Glucagon-like peptide-1; Mean HbA1c %, Mean value of glycated hemoglobin reported in percentage; Mean HbA1C mmol/mol (SD), Mean value of glycated hemoglobin reported in mmol/mol; SGLT2, Sodium-glucose cotransporter-2.

### 2.2 Whole exome sequencing

Genomic DNA was extracted from mononuclear cells using the Qiagen protocol. DNA purification was carried out with DNAeasy (Qiagen Inc, CA, USA) blood kit. Leukocytes were lysed using proteinase-K, and lysates were transferred to DNAeasy columns. Once DNA was captured in the columns, washes were performed with kit specific buffers to perform DNA elution. High quality, high molecular weight DNA was determined using NanoDrop2000 and TapeStation (Agilent Technologies, CA, USA).

The gDNA was shipped to the Genomics Core Lab of Tecnológico de Monterrey for WES. gDNA was quantified using Qubit dsDNA BR Assay Kit (Invitrogen, CA, USA). Quality was determined spectrophotometrically using Nanodrop One (Thermo Fisher Scientific, MA, USA). WES libraries were prepared using Illumina DNA Prep with Exome 1.0 Enrichment (Illumina, CA, USA) and quantified with Qubit dsDNA Assay (Invitrogen, CA, USA), library size was analyzed in S2 Standard DNA Cartridge for Sep 400, and sequencing performed in NovaSeq6000 (Illumina, CA, USA).

### 2.3 Computational bioinformatic and statistical analysis

Preprocessed sequences were aligned to human reference sequence (hg38) using Illumina-Dragen Enrichment pipeline. This pipeline was set to produce copy number variants (enable-cnv true). Binary Alignment Map (BAM) files resulting from the enrichment were removed from PCR duplicates using Picard Tools (http://broadinstitute.github.io/picard). Each BAM file was used to obtain somatic variants using GATK pipeline, and variants were annotated using ANNOVAR according to ClinVar database (reporting variant pathogenicity specific to MODY, and other metabolic diseases when no reports for MODY were available). We also assessed variant pathogenicity using the Monogenic Diabetes Expert Panel (https://clinicalgenome.org/affiliation/50016). We detailed the baseline allele frequency in Latin American individuals with mostly European and Native American Ancestry population according to ALFA allele frequency. No filters were used to remove benign variants, to explore the full spectrum of variants as there is limited data on variant pathogenicity in our population.

Variant Call Format (VCF) files were transformed and annotated using the vcf2maf tool and the Variant Effect Predictor 112 release database of ENSEMBL. In addition, the Maftools package version 2.21.1 in R version 4.4.1 was used to analyze and visualize the landscape of variants. Groups were constructed using read.maf and merge_maf functions. To compare groups mafCompare function was used with a Fisher test on a 2x2 contingency table and to detect genetic differences across groups. Graphs of mutations in genes of interest were constructed using Lollipop Plots to observe mutations by study subgroup using cBioPortal. We considered genetic variants with a frequency >75% across MODY patients and absent in T2DM and HC and used Web-gestalt for enrichment analysis (www.webgestalt.org).

We filtered all MODY 1–14 genetic variants to evaluate their frequency in every patient and group, excluding *PAX4*, *BLK*, *APPL1*, and *KLF11*, which have been disregarded as MODY causal genes. We tracked additional genes previously reported for their involvement in MODY. We conducted genetic analysis upon individualized cases to explore the association of variants per patient. We constructed a clustered heatmap on ClustVis (version 0.7.7) of MODY variants. Comparisons of genetic variant frequency across groups were conducted using Chi-square test. T-Student tests, and one-way ANOVA were used when applicable for comparing sociodemographic and clinical continuous variables. A p-value <0.05 was considered significant. Statistical package consisted of SPSS 24.0 (IBM, NY, USA).

### 2.4 Genetic ancestry analysis

To assess the genetic ancestry of MODY patients, we employed the EthSEQ R package, designed for ethnicity annotation from genotype data. High-quality, biallelic single nucleotide variants (SNVs) were extracted from each individual VCF file using bcftools, ensuring that only autosomal SNVs were retained. Variants with duplicate genomic positions were removed to avoid redundancy (16–18).

EthSEQ was run for each subject using the Gencode Exome reference model (hg38), which includes >1,500 individuals from diverse populations. The analysis was performed using ethseq. Analysis function with default parameters, enabling computation of a principal component analysis (PCA) and projection of each MODY sample into the reference space. Ancestry assignments were derived from proximity to reference clusters in the PCA space and visualized using EthSEQ (16–18).

## 3 Results

### 3.1 Baseline characteristics

A total of 51 patients were included in the study, 17 with MODY, 17 with T2DM, and 17 HC. The mean age at evaluation of MODY patients was 21.2 ± 5.7 years, 43.5 ± 10.1 years in T2DM patients, and 25.5 ± 3.4 years in HC. Mean age at diagnosis was 21.2 years, compared to 43.5 years in the T2DM group (p<0.001). Parental history of diabetes was present in 88% of MODY patients, and in 53% of T2DM subjects (p=0.023).

Considering the clinical presentation of MODY cases, *HNF1A*-MODY was the most frequently suspected subtype in 47% (8/17) of patients, followed by dual suspicion of *HNF1A-/HNF1B*-MODY in 22% of cases, *PAX4*-MODY (in ketosis-prone cases) in 11% of patients, and *GCK*-MODY, *HNF1B*-MODY, and *GCK/HNF1A*-MODY dual suspicion in single cases (**Table 1**). Regarding extra-pancreatic findings, one MODY patient presented gonadal dysgenesis. None of the MODY patients presented other clinical features such as deafness, renal cysts, liver abnormalities, or neurodevelopmental delays.

Mean HbA1c levels were 7.1% in MODY patients and 7.0% in T2DM patients. Regarding treatment goals, 47% of MODY cases and 53% of T2DM subjects reached target HbA1C levels (HbA1C <7%). BMI was significantly higher among patients with T2DM. The T2DM group exhibited the highest frequency of hypertension, whereas dyslipidemia was more prevalent among MODY patients. The mean Exeter score in MODY patients was 63.5 ± 14.3 (35.8-75.5%), while 4.6% in T2DM cases (**Table 1**).

### 3.2 Whole exome sequencing analysis reveals low utility of canonical diagnostic genes

**Figure 1:**
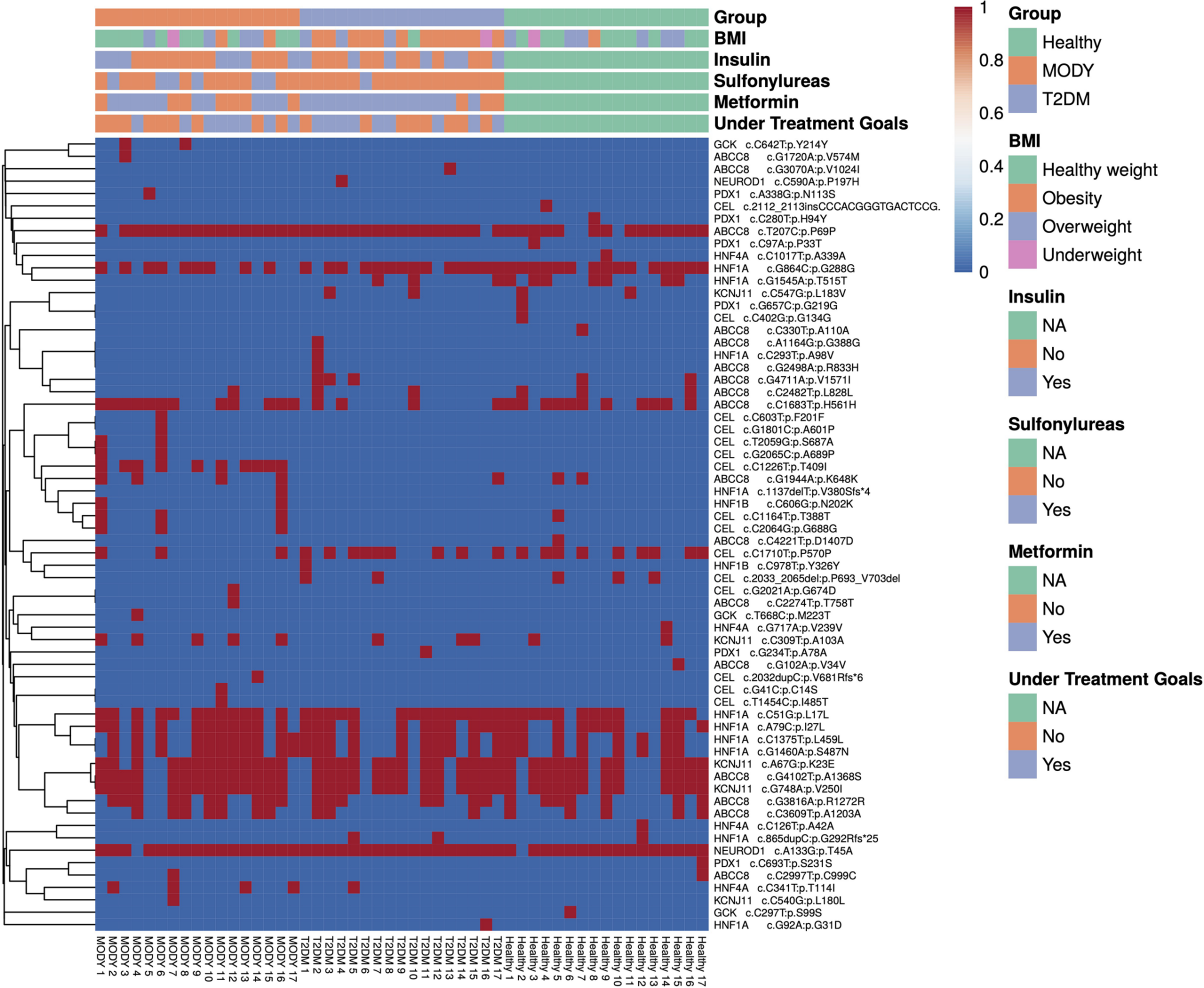
Heatmap of genetic variants of MODY-known genes. Figure shows a clustered heatmap of genetic variants of MODY-known genes across Mexican patients with MODY, T2DM, and healthy controls. BMI=Body Mass Index; Under Treatment Goals criteria was defined as a glycated hemoglobin equal or lower than 7.0%.

*ABCC8*, *CEL*, and *HNF1A* presented the highest number of variants, while no variants were identified in the *INS* gene (MODY 10), as displayed in **Tables 2**, **3**; **Figure 1**. The most relevant genes used for MODY diagnosis (e.g. *HNF4A*, *GCK*, *HNF1A*, *HNF1B*) presented similar variant frequencies across the three groups. In *HNF4A*, variant c.C341T:p.T114I (benign) was present in four (23.5%) MODY cases, one (5.9%) T2DM case, and absent in the HC (p=0.056). In *GCK*, variant c.C642T:p.Y214Y (benign/likely benign) was present in 11.8% of MODY cases, while absent in the rest of the groups (p=0.125). Variant c.T668C:p.M223T (unknown impact) was present in a single MODY case (5.9%) while absent in T2DM and HC (p=0.361) (**Table 2**).

**Table 2:** Clinical impact and frequency of genetic variants of MODY-known genes across groups.

Genetic variant	ClinGen expert panel	Clinical impact (ClinVar)	Condition reported	ALFA allele frequency in Latino population	MODY (n=17)		T2DM (n=17)		Healthy (n=17)		P-val
					n	%	n	%	n	%	
HNF4A (MODY 1)											
c.C341T:p.T114I	–	B	MODY	C= 0.95 T= 0.048	4	23.5	1	5.9	0	0.0	0.056
c.C1017T:p.A339A	–	LB	MODY	C= 1.0 T= 0.0	0	0.0	0	0.0	1	5.9	0.361
c.C126T:p.A42A	–	–	–	–	0	0.0	0	0.0	1	5.9	0.361
c.G717A:p.V239V	–	CIP	MODY	G= 1.0 A= 0.0	0	0.0	0	0.0	1	5.9	0.361
GCK (MODY 2)											
c.C297T:p.S99S	–	LB	MODY	–	0	0.0	0	0.0	1	5.9	0.361
c.C642T:p.Y214Y	–	B/LB	MODY	G= 0.97 A= 0.021	2	11.8	0	0.0	0	0.0	0.125
c.T668C:p.M223T	–	–	–	–	1	5.9	0	0.0	0	0.0	0.361
HNF1A (MODY 3)											
c.C51G:p.L17L	–	LB	HNF1A-related disorder	C=0.95 G=0.046 T=0.0	12	70.6	14	82.4	12	70.6	0.662
c.A79C:p.I27L	–	B	MODY	A=0.63 C=0.36	11	64.7	11	64.7	10	58.8	0.92
c.G864C:p.G288G	–	–	–	–	9	52.9	14	82.4	15	88.2	0.041
c.C1375T:p.L459L	–	B	MODY	C=0.63 T= 0.36	11	64.7	10	58.8	8	47.1	0.571
c.G1460A:p.S487N	–	B	MODY	G=0.63 A=0.36	11	64.7	10	58.8	8	47.1	0.571
c.A1741G:p.S581G	B	B	MODY	A=0.00 G=1.00	17	100	17	100	17	100	NA
c.C293T:p.A98V	–	B	MODY	C=0.98 T=0.02	0	0.0	1	5.9	0	0.0	0.361
c.865dupC:p.G292Rfs*25	P	P	MODY	CCCCCCCC=1.000 CCCCCCCCC = 0.000	0	0.0	2	11.8	1	5.9	0.346
c.G1545A:p.T515T	–	B	MODY	G=0.94 A=0.057 T=0.00	0	0.0	3	17.6	7	41.2	0.01
c.G92A:p.G 31D	–	B/LB	MODY	G=1.0 A=0.0 C=0.0	0	0.0	1	5.9	0	0.0	0.361
c.1137delT:p.V380Sfs*4	P	P	MODY	–	1	5.9	0	0.0	0	0.0	0.361
PDX 1 (MODY 4)											
c.G234T:p.A78A	–	LB	MODY	–	0	0.0	1	5.9	0	0.0	0.361
c.G657C:p.G219G	–	–	–	–	0	0.0	0	0.0	1	5.9	0.361
c.C97A:p.P33T	–	LB/VUS	MODY	C=1.0 A=0.0 G=0.0 G=0.0	0	0.0	0	0.0	1	5.9	0.361
c.C280T:p.H94Y	–	VUS	MODY	–	0	0.0	0	0.0	1	5.9	0.361
c.C693T:p.S231S	–	LB	MODY	C=1.0 G=0.0 T=0.0	0	0.0	0	0.0	1	5.9	0.361
c.A338G:p.N113S	–	VUS	MODY	–	1	5.9	0	0.0	0	0.0	0.361
HNF1B (MODY 5)											
c.C978T:p.Y326Y	–	–	–	–	0	0.0	1	5.9	0	0.0	0.361
c.C606G:p.N202K	–	B	MODY	–	2	11.8	0	0.0	0	0.0	0.125
NEUROD1 (MODY 6)											
c.A133G:p.T45A	–	B	MODY	T=0.25 C=0.74	16	94.1	17	100	16	94.1	0.346
c.C590A:p.P197H	–	B	MODY	G=0.98 T=0.018	0	0.0	1	5.9	0	0.0	0.361
CEL (MODY 8)											
c.C1710T:p.P570P	–	B/LB	NS	C=0.978 G=0.0 T=0.022	3	17.6	9	52.9	9	52.9	0.054
c.2033_2065del:p.P693_V703del	–	–	–	–	0	0.0	2	11.8	3	17.6	0.212
c.C402G:p.G134G	–	B/LB	MODY	C=1.0 G=0.0 T=0.0	0	0.0	0	0.0	1	5.9	0.361
c.2112_2113insCCCACGGGTGACTCCGAGACCGCCCCCGTGCCG:p.S720_G721insETAPVPPTGDS	–	–	–	–	0	0.0	0	0.0	1	5.9	0.361
c.C1164T:p.T388T	–	B	NS	–	3	17.6	0	0.0	1	5.9	0.15
c.C2064G:p.G688G	–	B	NS	C=1.0 G=0.0	3	17.6	0	0.0	0	0.0	0.041
c.C603T:p.F201F	–	–	–	–	1	5.9	0	0.0	0	0.0	0.361
c.C1226T:p.T409I	–	B	NS	–	10	58.8	0	0.0	0	0.0	<0.001
c.T2059G:p.S687A	–	–	–	–	2	11.8	0	0.0	0	0.0	0.125
c.G2065C:p.A689P	–	VUS	MODY	–	2	11.8	0	0.0	0	0.0	0.125
c.G1801C:p.A601P	–	LB	NS	–	1	5.9	0	0.0	0	0.0	0.361
c.G41C:p.C14S	–	–	–	–	1	5.9	0	0.0	0	0.0	0.361
c.T1454C:p.I485T	–	B / LB	MODY	T=0.948 C=0.052	1	5.9	0	0.0	0	0.0	0.361
c.G2021A:p.G674D	–	–	–	–	1	5.9	0	0.0	0	0.0	0.361
c.2032dupC:p.V681Rfs*6	–	VUS	MODY	CCCCCCCCC=1.000 CCCCCCCC = 0.000, CCCCCCCCCC = 0.000	1	5.9	0	0.0	0	0.0	0.361
INS (MODY 10)											
No variants found	–	–	–	–	0	0	0	0	0	0	NA
ABCC8 (MODY 12)											
c.T207C:p.P69P	–	B	Neonatal diabetes	A=0.44 G=0.55 T=0.0	16	94.1	16	94.1	14	82.4	0.412
c.G4711A:p.V1571I	–	–	–	–	0	0.0	3	17.6	2	11.8	0.212
c.G4102T:p.A1368S	–	–	–	–	14	82.4	13	76.5	12	70.6	0.721
c.G3816A:p.R1272R	–	B	Neonatal diabetes	C=0.62 T=0.37	10	58.8	7	41.2	7	41.2	0.492
c.C3609T:p.A1203A	–	LB	NS	G=1.0 A=0.0 C=0.0	7	41.2	3	17.6	5	29.4	0.322
c.G2498A:p.R833H	–	–	–	–	0	0.0	1	5.9	0	0.0	0.361
c.C2482T:p.L828L	–	B	Neonatal diabetes	G=0.94 A=0.05	1	5.9	2	11.8	3	17.6	0.567
c.C1683T:p.H561H	–	–	–	–	12	70.6	4	23.5	11	64.7	0.011
c.A1164G:p.G388G	–	–	–	–	0	0.0	1	5.9	0	0.0	0.361
c.G3070A:p.V1024I	–	–	–	–	0	0.0	1	5.9	0	0.0	0.361
c.G1944A:p.K648K	–	VUS/ B	T2DM Neonatal diabetes	C=0.87 T=0.12	4	23.5	1	5.9	2	11.8	0.314
c.C4221T:p.D1407D	–	–	–	–	0	0.0	0	0.0	1	5.9	0.361
c.C330T:p.A110A	–	B	T2DM Neonatal diabetes	G=0.98 A=0.01	0	0.0	0	0.0	1	5.9	0.361
c.G102A:p.V34V	–	VUS	MODY	C=0.99 T=0.006	0	0.0	0	0.0	1	5.9	0.361
c.C2997T:p.C999C	–	VUS	MODY	G=0.99 A=0.007	1	5.9	0	0.0	1	5.9	0.594
c.G1720A:p.V574M	–	VUS	NS	–	1	5.9	0	0.0	0	0.0	0.361
c.C2274T:p.T758T	–	–	–	–	1	5.9	0	0.0	0	0.0	0.361
KCNJ11 (MODY 13)											
c.G748A:p.V250I	–	LB	NS	–	14	82.4	13	76.5	12	70.6	0.721
c.A67G:p.K23E	–	–	–	–	13	76.5	13	76.5	12	70.6	0.902
c.C547G:p.L183V	–	–	–	–	0	0.0	2	11.8	2	11.8	0.338
c.C309T:p.A103A	–	LB	MODY	–	5	29.4	3	17.6	2	11.8	0.419
c.C540G:p.L180L	–	B	MODY	–	1	5.9	0	0.0	0	0.0	0.361

VUS, Variant of Uncertain Significance; P, Pathogenic; LP, Likely Pathogenic; B, Benign; LB, Likely Benign; CIP, Conflicting interpretation of pathogenicity; NS, Not Specified; “-”, Not Available.

**Table 3:** Number of detected genetic variants in MODY-known genes among all subjects.

			Mody	1	2	3	4	5	6	8	10	12	13
Pt. #	Susp MODY	Tx	Exeter (%)	HNF 4A	GCK	HNF 1A	PDX1	HNF 1B	NEUROD1	CEL	INS	ABCC8	KCNJ11
MODY group													
1	3	I	45.5	0	0	4	0	1	1	6	0	4	3
2	3/5	I, M, G	75.5	1	0	5	0	0	1	0	0	3	2
3	3/5	I, M, D, Lg	75.5	0	1	2	0	0	1	1	0	5	1
4	3	M	75.5	0	1	5	0	0	0	1	0	6	3
5	9	M, Ln	45.5	0	0	2	1	0	1	0	0	2	0
6	5	M, G	62.4	0	0	6	0	0	1	8	0	2	0
7	3	S, P, G	75.5	1	0	2	0	0	1	0	0	6	3
8	9	D&E	75.5	0	1	2	0	0	1	0	0	4	2
9	3	Lg, M, G	35.8	0	0	6	0	0	1	1	0	2	3
10	3	M	75.5	0	0	6	0	0	1	0	0	4	2
11	3	I	45.5	0	0	5	0	0	1	3	0	6	2
12	2	I	75.5	0	0	5	0	0	1	1	0	5	3
13	3/5	I, Ln	75.5	1	0	6	0	0	1	1	0	2	2
14	3/5	D, M, G	58.0	0	0	5	0	0	1	2	0	4	2
15	3	M, G, Ln, D	75.5	0	0	2	0	0	1	1	0	5	2
16	3	M	75.5	0	0	6	0	1	1	4	0	5	3
17	2/3	I	49.4	1	0	3	0	0	1	0	0	2	0
Type 2 diabetes group													
1	–	I, M, D, Ln	–	0	0	6	0	0	1	1	0	1	0
2	–	M, Ln, Lg	–	0	0	5	0	0	1	0	0	6	2
3	–	M, P, Lg	–	0	0	6	0	0	1	1	0	4	2
4	–	M	–	0	0	3	0	0	1	0	0	4	2
5	–	I, M, P, D	–	1	0	5	0	0	1	1	0	2	2
6	–	M, Lg, D, Gc	–	0	0	2	0	0	1	1	0	1	0
7	–	M, Lg, D	–	0	0	2	0	0	1	1	0	2	3
8	–	I, M, Ln	–	0	0	2	0	0	1	1	0	2	2
9	–	M	–	0	0	6	0	0	1	0	0	2	2
10	–	M, D, Sit	–	0	0	3	0	0	1	0	0	3	0
11	–	I, M, Lg	–	0	0	6	0	0	1	0	0	3	2
12	–	M	–	0	0	5	0	0	1	1	0	3	2
13	–	I, M, Lg	–	0	0	6	0	0	1	0	0	1	0
14	–	I, D, Ln	–	0	0	3	0	0	1	1	0	2	3
15	–	M, Lg	–	0	0	6	0	0	1	0	0	4	3
16	–	D, Sit	–	0	0	4	0	0	1	0	0	2	2
17	–	I, D, Lg	–	0	0	6	0	0	1	1	0	4	2
Healthy control group													
1	–	–	–	0	0	6	0	0	1	0	0	5	2
2	–	–	–	0	0	6	0	0	0	1	0	3	0
3	–	–	–	0	0	3	0	0	1	0	0	2	3
4	–	–	–	0	0	3	0	0	1	1	0	4	2
5	–	–	–	0	0	6	0	0	1	2	0	5	2
6	–	–	–	0	0	2	0	0	1	0	0	4	2
7	–	–	–	0	0	5	0	0	1	1	0	4	2
8	–	–	–	0	0	4	0	0	1	0	0	1	0
9	–	–	–	0	0	4	0	0	1	0	0	5	2
10	–	–	–	0	0	6	0	0	1	1	0	1	2
11	–	–	–	0	0	2	0	0	1	0	0	1	0
12	–	–	–	0	0	3	0	0	1	1	0	2	0
13	–	–	–	0	0	2	0	0	1	1	0	2	0
14	–	–	–	0	0	6	0	0	1	0	0	3	3
15	–	–	–	0	0	6	0	0	1	0	0	4	2
16	–	–	–	0	0	3	0	0	1	1	0	4	2
17	–	–	–	0	0	3	0	0	1	1	0	5	2

Pt. #, Patient number; Susp. MODY, Suspected MODY type; Tx, treatment; I, Insulin; M, Metformin; D, Dapagliflozin; Lg, Liraglutide; Ln, Linagliptin; Sit, Sitagliptin; D&E, Diet and exercise; G, Glimepride; Gc, Glibenclamide; P, Pioglitazone; NA, Not apply; MODY 2, GCK-MODY; MODY 3, HNF1A-MODY; MODY 5, HNF1B-MODY; MODY 9, PAX4-MODY.

In *HNF1A*, the pathogenic variant c.1137delT:p.V380Sfs*4 was only present in a single MODY case (5.9%) (p=0.361), while another pathogenic *HNF1A* variant (c.865dupC:p.G292Rfs*25) was detected in two individuals with T2DM and one HC, while absent in the MODY group (**Table 2**). Several variants in this gene presented similar frequencies across groups including c.A1741G:p.S581G (benign), c.C1375T:p.L459L (benign), c.C51G:p.L17L (likely benign), and c.G864C:p.G288G (unknown impact).

We compared variant frequencies of other canonical MODY genes. We only found significant differences in *CEL* and *ABCC8*. In *CEL*, variant c.C1226T:p.T409I (unknown impact) (p=0.001), was exclusively present in 10 MODY cases and variant c.C2064G:p.G688G (unknown impact) (p=0.041) was only present in 3 (17.6%) MODY cases. The benign variant c.C1683T:p.H561H in *ABCC8* gene was present in 12 patients with MODY (70.6%), 4 patients with T2DM (23.5%), and 11 HC (64.7%) (p=0.011). **Supplementary Table 1** shows all identified variants with greater detail.

We analyzed other MODY-associated genes reported in other populations (*RFX6*, *NKX6-1*, *AKT2*, *NKX2-2*, *PCBD1*, *MTOR*, *TBC1D4*, *CACNA1E*, and *MNX1*) and found no significant differences across groups (**Supplementary Table 2**).

### 3.3 Whole exome sequencing analysis of MODY, T2DM and HC reveals potential differential diagnostic genes

We searched for differential genetic variant enrichment across groups identifying 14 genes and a total of 26 variants detected in 75-100% of MODY patients, that were absent in patients with T2DM and HC (**Table 4**; **Supplementary Table 3**). These genes include *MAP2K*, *TPTE*, *SYT15*, *PEX5*, *KCNJ12*, *KTM2C*, *OR2A1*, *RIMBP3*, *TRIM49C*, *AQP12B*, *OR51A4*, *RIMBP3B*, *ZNF717*, and *SUSD2*.

**Table 4:** Variant frequency of novel candidate genes with enrichment in the MODY group.

Genetic variant	MODY (n=17)		T2DM ( n=17)		Healthy (n=17)		All groups
	n	%	n	%	n	%	P-value
Genetic variants enriched in the MODY group							
MAP2K3							
c.C118A:p.P40T	16	94.1	0	0.0	0	0.0	<0.001
c.G164C:p.R55T	17	100	0	0.0	0	0.0	<0.001
c.C304T:p.Q102Ter	17	100	0	0.0	0	0.0	<0.001
c.G281T:p.R94L	17	100	0	0.0	0	0.0	<0.001
c.C286T:p.R96W	17	100	0	0.0	0	0.0	<0.001
PEX5							
c.210+77_210+121delCAGCCTCTGAGGCAGTGAGTGTTCTTGAGGTGGAAAGCCCAGGTG_	16	94.1	0	0.0	0	0.0	<0.001
ZNF717							
c.1729delA;p.T577ProfsTer51	13	76.5	0	0.0	0	0.0	<0.001
c.G1736A:p.R579H	13	76.5	0	0.0	0	0.0	<0.001
c.G1575T:p.K525N	13	76.5	0	0.0	0	0.0	<0.001
c.C1567T:p.H523Y	13	76.5	0	0.0	0	0.0	<0.001
c.G2551C:p.E851Q	13	76.5	0	0.0	0	0.0	<0.001
KMT2C							
c.2447dupA:p.Y816Ter	13	76.5	0	0.0	0	0.0	<0.001
TPTE							
c.A1156G:p.K386E	17	100	0	0.0	0	0.0	<0.001
c.A1445C:p.Y482S	16	94.1	0	0.0	0	0.0	<0.001
KCNJ12							
c.G128A:p.R43H	17	100	0	0.0	0	0.0	<0.001
OR2A1							
c.C220T:p.R74C	17	100	0	0.0	0	0.0	<0.001
c.C503G_p.S168C	16	94.1	0	0.0	0	0.0	<0.001
c.A217G:p.T73A	16	94.1	0	0.0	0	0.0	<0.001
c.C470T:p.A157V	13	76.5	0	0.0	0	0.0	<0.001
RIMBP3							
c.T3273G:p.D1091E	14	82.4	0	0.0	0	0.0	<0.001
TRIM49C							
c.C877G:p.H293D	16	94.1	0	0.0	0	0.0	<0.001
AQP12B							
c.A46G:p.T16A	16	94.1	0	0.0	0	0.0	<0.001
OR51A4							
c.C863T:p.T288M	17	100	0	0.0	0	0.0	<0.001
SYT15							
c.G927C:p.E309D	14	82.4	0	0.0	0	0.0	<0.001
RIMBP3B							
c.C4537T:p.R1513C	16	94.1	0	0.0	0	0.0	<0.001
SUSD2							
c.G101A:p.R34H	16	94.1	0	0.0	0	0.0	<0.001

**Figure 2:**
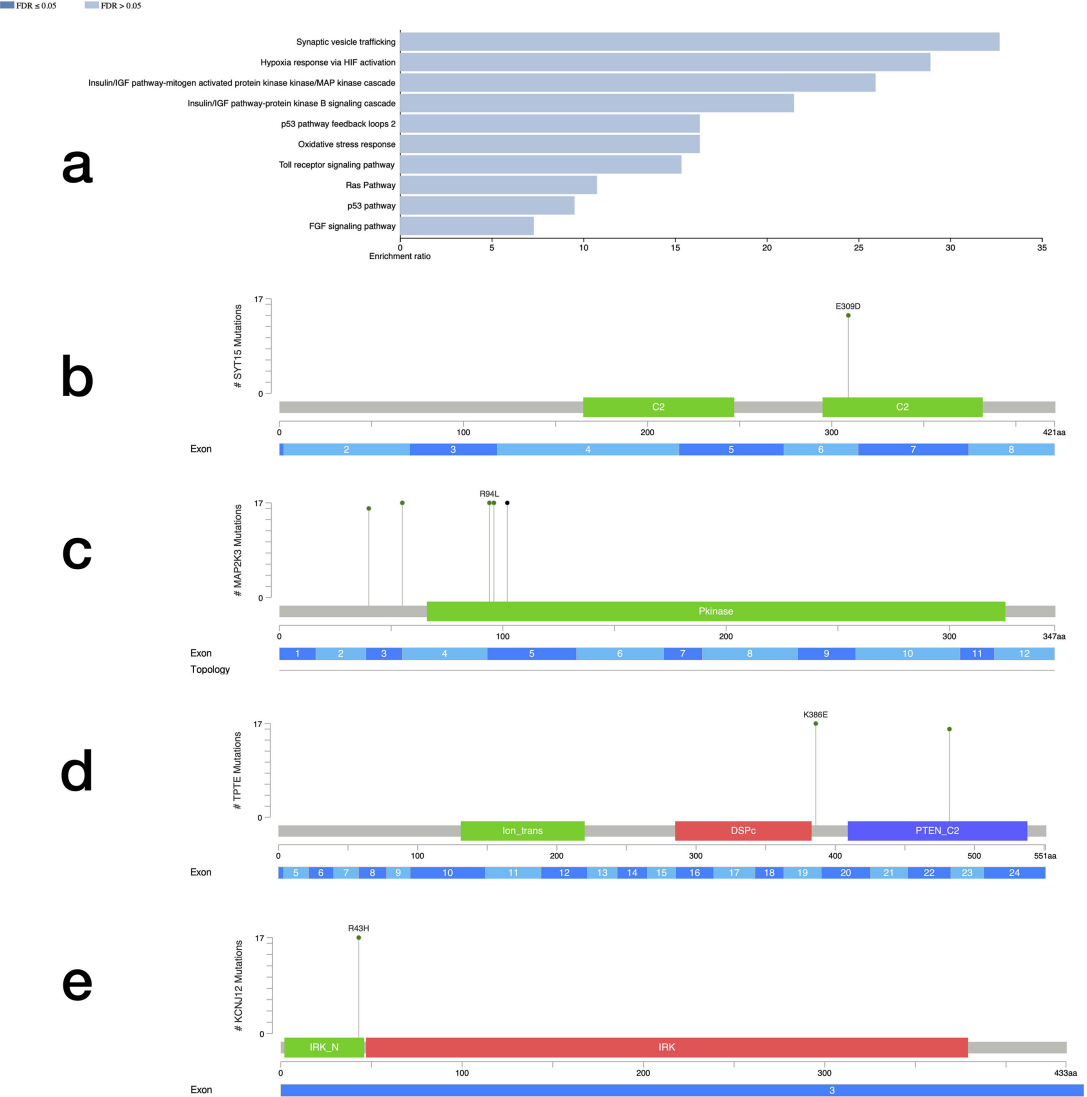
Enrichment analysis and lollipop diagrams of proposed genes of interest. Figure shows the enrichment analysis of MODY candidate genes highlighting key pathways of glucose metabolism **(A)** and lollipop diagrams of SYT15 **(B)**, MAP2K3 **(C)**, TPTE **(D)**, and KCNJ12 **(E)** genes.

Several *MAP2K3* genetic variants were present in 100% of MODY cases but absent in patients with T2DM and HC. These include c.G281T:p.R94L, c.C286T:p.R96W, c.C304T:p.Q102Ter, and c.G164C:p.R55T. Variant c.G164C:p.R55T was exclusively found in 94% of MODY patients (**Figure 2**).

Regarding *TPTE* gene, variants c.A1156G:p.K386E, and c.A1445C:p.Y482S were found in 100% and 94% of MODY cases, respectively, while they were absent in T2DM and HC. The *KCNJ12* c.G128A:p.R43H variant was exclusively present in 100% of MODY cases. Additional variants found in 100% of MODY cases while absent in T2DM and HC included *OR51A4* c.C863T:p.T288M, *OR2A1* c.C220T:p.R74C, and *SYT15* c.G927C.p:E309D (**Figure 2**). Additionally, in 94% of patients with MODY but in none of the patients with T2DM or HC, we identified a splicing site variant in *PEX5*.

According to enrichment analysis, identified genes are involved in synaptic vesicle trafficking (*SYT15*), insulin/IGF pathway-mitogen activated protein kinase kinase/MAP kinase cascade (*MAP2K3*), insulin/IGF pathway-protein kinase B (AKT) signaling cascade (*TPTE*), oxidative stress response (*MAP2K3*), among others (**Figure 2**).

### 3.4 Ancestry and ethnicity analyses across MODY, T2DM, and HC groups

We inferred individual ancestry coefficients using the snmf function from the LEA R package, selecting K = 2 based on cross-entropy minimization. The resulting PCA plot (**Supplementary Figure 1A**) revealed a clear genetic structure, with most individuals showing dominant ancestry from one of the two inferred clusters. Most MODY patients clustered together, suggesting a common allelic structure among them, while T2DM and HC clustered together. Thus, an ancestry component may represent a broader ancestral background in the MODY cohort. This structure may reflect underlying demographic patterns that could influence MODY diagnosis or prevalence (**Supplementary Figure 1B**).

Ethnicity analysis revealed that most subjects presented mixed ethnic genetic backgrounds. Four MODY patients and a T2DM patient could not be correctly assigned. Most MODY patients showed diverse genetic backgrounds, resulting from admixture of American, European (EUR), African (AFR), South Asian (SAS), and Eastern Asian (EAS) backgrounds. However, four MODY cases showed only EUR and AMR ethnicity. Most T2DM and HC showed five ethnic backgrounds as well (**Supplementary Figure 1B**).

## 4 Discussion

In the Mexican population, the prevalence of variants in MODY-known genes is similar in patients with MODY, T2DM, and HC. Only one patient with MODY (5.9%) presented a pathogenic variant in *HNF1A* (c.1137delT:p.V380Sfs*4), which was absent in patients with T2DM and HC. Most Mexican patients with MODY did not harbor any pathogenic variant in MODY canonical genes. Notably, two patients with T2DM and a HC presented a pathogenic variant in *HNF1A* (c.865dupC:p.G292Rfs*25), which was absent in MODY patients. *HNF1A* has been proposed to be involved in the pathogenesis of T2DM in Latino populations (19). Other MODY-known genes such as *CEL*, *KCNJ11*, and *ABCC8*, presented similar variant frequencies across groups, while two variants in *CEL* and one in *ABCC8* displayed significant differences. Remarkably, variant c.C1226T:p.T409I in *CEL* was detected in 58% of patients with MODY but in none of the HC or individuals with T2DM. Our data underscores the complex genomic architecture of MODY and supports the notion that in certain populations, this condition is not caused by a single genetic defect (20).

Our data suggests that MODY-known genes are insufficient for the establishment of a definitive genetic diagnosis in most cases in the Mexican population, while several aspects must be evaluated when considering genetic variants, such as the baseline frequency in the healthy population, pathogenicity assessment tools based on distinct populations, variable penetrance, and interaction between variants.

Patients with MODY presented a high degree of allelic heterogeneity when analyzed individually as they all displayed different variants among canonical genes, suggesting the notion that in some populations MODY could behave in a polygenic manner. Concomitant variants could contribute to MODY phenotypical presentation through the interplay of polygenic backgrounds over isolated variants. In a recent GWAS meta-analysis comprising over 51,000 patients with T2DM, genetic variants in MODY genes were found to be associated with a 4-to 8-fold increased diabetes risk (21). Other authors have pointed at MODY polygenic traits given the existence of variants with low-penetrance and limited-pathogenicity (21, 22). Additionally, epigenetic and non-genetic factors should be considered, including environmental conditions, and ethnogenetic background, especially in patients who fulfill diagnostic criteria but lack a genetic diagnosis (22). The consideration of these aspects, in addition to the presence of multiple variants in MODY-known genes and other genes possibly involved in other glycemic regulation pathways, challenges the consideration of MODY as an exclusively monogenic disease. In fact, a recent study described the contribution of a polygenic background and common genetic variants into MODY clinical presentation (20).

The absence of variants of clinical significance in MODY canonical genes in most patients, and the fact that these genes were mainly described in Caucasian populations, prompted us to investigate the presence of other genetic variants that could explain the MODY genetic landscape in the Mexican population. Several authors have described other genes involved in early onset diabetes in non-Caucasian populations such as *RFX6* p.Hi93Leu variant in Finnish MODY cases, *NKX6-1*, and *AKT2* in South Indian patients with MODY and *MTOR*, *TBC1D4*, *CACNA1E*, and *MNX1* in Polish patients (8, 9, 22–26). Moreover, the role of *PCBD1* mutations in early-onset non-autoimmune diabetes has been described (25, 26). We searched for variants in these genes and did not find differences across groups.

While underrepresented in genomics research, Latin American populations are genetically heterogeneous, having distinct ancestral compositions resulting from continental admixture, population bottlenecks, and historical migration (27). Latin America represents a crucial context to study diabetes genetics due to the disproportionately heavy disease burden in this population (28, 29). We propose 14 genes of interest significantly enriched in Mexican patients with MODY. These could serve as diagnostic biomarkers to better identify MODY patients, differentiating them from those with T2DM.

**Figure 3:**
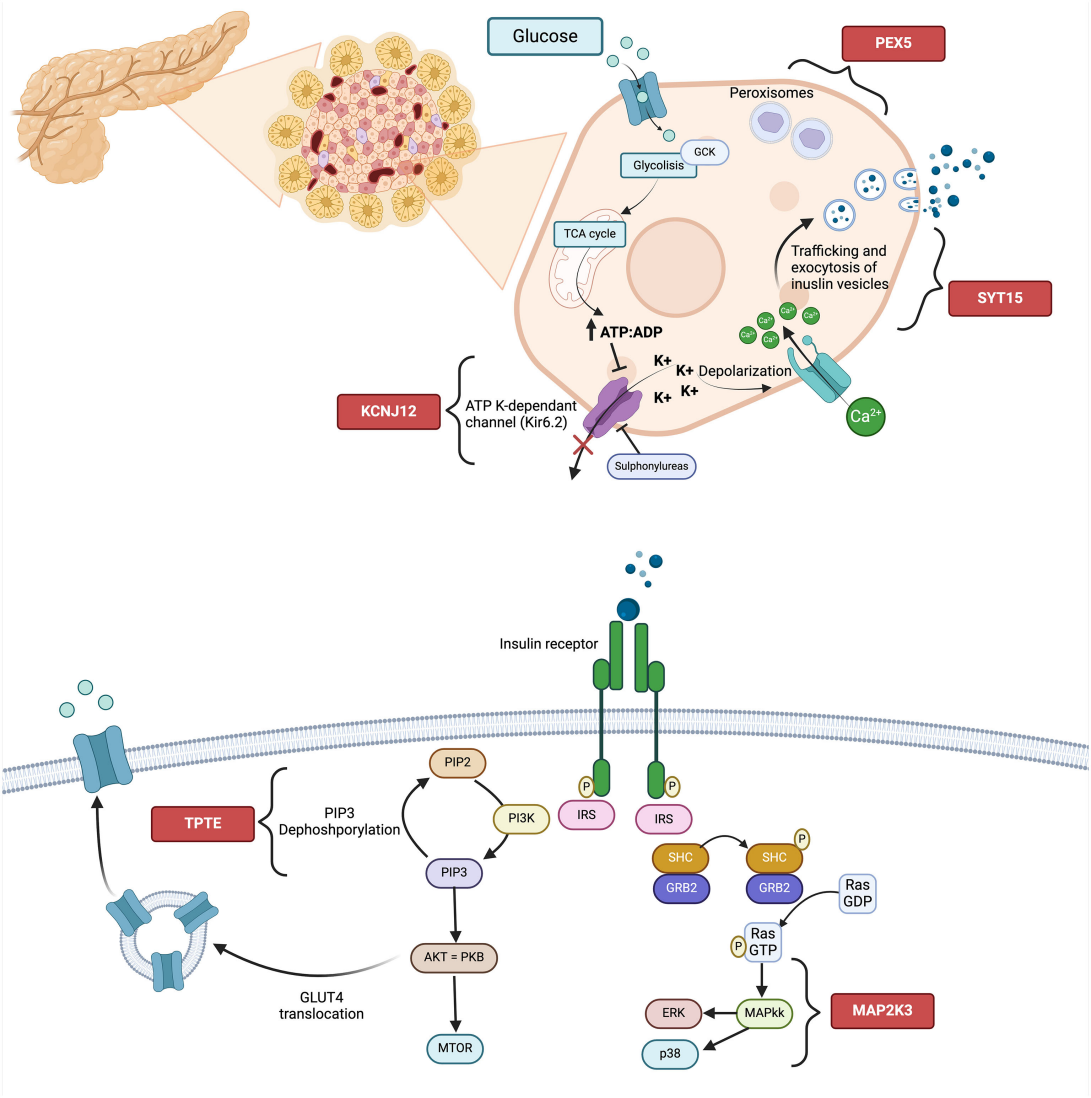
Potential sites of involvement of genes of interest. Figure shows potential sites in insulin release and glucose metabolism pathways where proposed genes of interest could be involved.

Enrichment analysis revealed the proposed genes to be involved in several metabolic and signaling pathways such as synaptic vesicle trafficking (*SYT15*), hypoxia response via Hypoxia Inducible Factor activation, insulin/IGF pathway-mitogen activated protein kinase kinase/MAP kinase cascade (*MAP2K3*), insulin/IGF pathway-protein kinase B signaling cascade (*TPTE*), and oxidative stress response. Several of the proposed genes could have relevant implications for glucose metabolism and in beta-cell function (**Figure 3**).

### 4.1 Potential metabolic involvement of proposed genes

Physiologically, after preproinsulin is synthesized in the endoplasmic reticulum of pancreatic beta-cells, proinsulin is directed to the Golgi apparatus and packaged into secretory granules (30). Beta-cell glucose metabolism deactivates the ATP-sensitive K-channels (31). Increased intracellular potassium induces membrane depolarization and increased calcium influx, promoting vesicle transport to release insulin. Insulin binds to the insulin receptor (IR), inducing recruitment of insulin receptor substrate (IRS) to activate two main pathways: PI3K-Akt and MAPK (31). Firstly, IRS activates phosphoinositide 3-kinase (PI3K), which phosphorylates phosphatidylinositol 4,5-bisphosphate (PIP2) into phosphatidylinositol 3,4,5-triphosphate (PIP3) to activate downstream effectors like AKT/PKB and mTOR to promote GLUT4 translocation (31). Second, IRS induces phosphorylation of Ras-GDP to Ras-GTP. Then, downstream effectors such as MAP kinase kinase (MAPkk) activate ERK1/2 to promote GLUT-4 transcription (31).

Synaptic vesicle trafficking is crucial for insulin release. The *SYT15* gene encodes a membrane trafficking protein belonging to the synaptotagmin family (32). Synaptotagmins are calcium sensors that regulate granule fusion to the plasma membrane (33). Various synaptotagmin family members modulate beta-cell maturation, calcium sensitivity, and vesicle release. Specifically, synaptotagmin-7 null mice present altered insulin secretion (32, 34).

The transmembrane phosphatase with tensin homolog (*TPTE*) gene encodes a PTEN-related tyrosine phosphatase with key roles in endocrine and testicular signal transduction pathways (32). In the insulin signaling pathway, PTEN (Phosphatase and tensin homolog) exerts negative regulation, as dephosphorylates PIP3 into PIP2 in the PI3K pathway, reducing insulin response (31). Thus, loss-of-function mutations could alter insulin signaling (31).

The *KCNJ12* gene, similarly to *KCNJ11* (MODY 13 gene) encodes a potassium inwardly rectifying channel (Kir 2.2) (32). Inhibition of potassium influx is crucial for beta-cell depolarization and insulin secretion. *KCNJ12* acts as a target for miR-132-3p to modulate the AKT signaling pathway, involved in glucose metabolism by increasing GLUT-1/4 translocation (35).

The *MAP2K3* gene encodes a protein belonging to the MAPkk family, which mediates signaling through phosphorylation of MAP14/p38-MAPK (32). In glycemic regulation, *MAP2K3* activity is enhanced by insulin, which is necessary for adequate expression of glucose transporters (36). Li et al. evaluated the effect of AMPK (AMP-activated protein kinase) in p38-MAPK activation, being involved in glucose transport, while its inhibition reduced GLUT-4 translocation (37). Other authors described altered p38-MAPK activity in T2DM patients’ skeletal muscle and adipose tissue (38).

The *PEX5* gene plays a key role in peroxisomal protein import, as its product binds to the C-terminal PTS1-type tripeptide peroxisomal targeting signal (32). *PEX5* delivers folded proteins from the cytosol into peroxisomes, translocating them across the membrane and returning them to the cytosol (39). Rip-Pex5/- mice present impaired insulin secretion (39). Peroxisomes are involved in lipid metabolism, and beta-cell homeostasis, while peroxisome proliferator-activated receptor alpha agonists confer protection to beta-cells against fatty acids (40).

Study strengths include conducting WES unlike studies using panels with a limited number of genes, allowing a comprehensive evaluation of genetic variants of non-canonical genes and comparison of our findings with HC and T2DM patients of the same ethnogenetic background. Limitations include the need for external validation of candidate genes, a small simple size, and lack of genetic evaluation of MODY patient relatives. Future perspectives include performing external validation of the candidate genes, functional analysis to determine causality, genotype-phenotype association analysis, and creation of polygenic risk scores. If further validated in external cohorts, the proposed candidate variants could help incorporate new tools into clinical practice to diagnose MODY in Latino populations and to better understand glycemic regulation mechanisms.

## 5 Conclusion

This represents one of the first efforts to characterize the genomic landscape of MODY in the Mexican population though WES, demonstrating that genes routinely used for diagnosis present similar frequencies in MODY patients when compared to T2DM and HC cases. Identified genes may serve as MODY diagnostic biomarkers in Latino populations.

## Data Availability

The datasets presented in this study can be found in online repositories. The names of the repository/repositories and accession number(s) can be found below: https://www.ncbi.nlm.nih.gov/, PRJNA1457790.
